# Genome-wide distribution of histone H4 Lysine 16 acetylation sites and their relationship to gene expression

**DOI:** 10.1186/2041-9414-4-3

**Published:** 2013-04-12

**Authors:** Nobuo Horikoshi, Pankaj Kumar, Girdhar G Sharma, Min Chen, Clayton R Hunt, Kenneth Westover, Shantanu Chowdhury, Tej K Pandita

**Affiliations:** 1 Department of Radiation Oncology, Division of Molecular Radiation Biology, University of Texas Southwestern Medical Center, Dallas, TX 75390, USA; 2 Department of Clinical Sciences, UT Southwestern Medical Center, Dallas, TX 75390, USA; 3 Department of Radiation Oncology, Washington University School of Medicine, St. Louis, MO, 63108, USA; 4 G.N.R. Center for Genome Informatics Unit, CSIR- Institute of Genomics and Integrative Biology, Delhi, 110007, India; 5 G.N.R. Center for Genome Informatics and Proteomics and Structural Biology Unit, CSIR-Institute of Genomics and Integrative Biology, Mall Road, Delhi 110007, India

**Keywords:** Genome, H4K16ac, Gene expression

## Abstract

**Background:**

Histone post-translational modifications are critical determinants of chromatin structure and function, impacting multiple biological processes including DNA transcription, replication, and repair. The post-translational acetylation of histone H4 at lysine 16 (H4K16ac) was initially identified in association with dosage compensation of the Drosophila male X chromosome. However, in mammalian cells, H4K16ac is not associated with dosage compensation and the genomic distribution of H4K16ac is not precisely known. Therefore, we have mapped the genome-wide H4K16ac distribution in human cells.

**Results:**

We performed H4K16ac chromatin immunoprecipitation from human embryonic kidney 293 (HEK293) cells followed by hybridization to whole-genome tiling arrays and identified 25,893 DNA regions (false discovery rate <0.005) with average length of 692 nucleotides. Interestingly, although a majority of H4K16ac sites localized within genes, only a relatively small fraction (~10%) was found near promoters, in contrast to the distribution of the acetyltransferase, MOF, responsible for acetylation at K16 of H4. Using differential gene expression profiling data, 73 genes (> ±1.5-fold) were identified as potential H4K16ac-regulated genes. Seventeen transcription factor-binding sites were significantly associated with H4K16ac occupancy (p < 0.0005). In addition, a consensus 12-nucleotide guanine-rich sequence motif was identified in more than 55% of the H4K16ac peaks.

**Conclusions:**

The results suggest that H4K16 acetylation has a limited effect on transcription regulation in HEK293 cells, whereas H4K16ac has been demonstrated to have critical roles in regulating transcription in mouse embryonic stem cells. Thus, H4K16ac-dependent transcription regulation is likely a cell type specific process.

## Background

Covalent modifications of histone tails are thought to alter chromatin structure, thereby controlling DNA transcription, repair and replication. Acetylation of lysine 16 of histone H4 (H4K16ac) has the potential to create or secure binding platforms for transcriptional factors as well as other chromatin-modifying enzymes [1,2]. Histone H4 lysine 16 is acetylated by males absent on the first (MOF, also called MYST1 or KAT8), a highly conserved member of the MYST histone acetyltransferase (HAT) family. MOF itself was originally discovered in Drosophila as an essential component of the X chromosome dosage compensation complex (DCC), also known as the male-specific lethal (MSL) complex. MOF increases expression of X-linked genes in male flies by 2-fold [3-6] and disruption of the MOF chromobarrel domain leads to genome-wide H4K16ac loss and compromised MSL targeting to X-linked genes [7]. In addition to its well defined role in dosage compensation, MOF has recently been found at active promoters genome-wide in both male and female flies, where it is bound as part of the nonspecific lethal (NSL) complex [8-10]. Other members of the MYST acetyltransferase family including acute myeloid leukemia (MOZ), transcriptional silencing in *Saccharomyces cerevisiae* (SAS2 and YBF2/SAS3), interactions with human immunodeficiency virus Tat in humans (TIP60), are also known to have transcriptional functions as well as roles in DNA damage repair [11-19].

In mammals, MOF is essential for development. Constitutive ablation of *Mof* leads to peri-implantation embryonic lethality in mice [20,21]. MOF is also essential for post-mitotic cell survival as Cre-mediated conditional MOF deletion in Purkinje cells induces chromatin blebbings and cell death, suggesting Mof has a crucial role in maintenance of chromatin structures in vivo [22]. Histone acetylation has been suggested to have a role in both transcriptional initiation and elongation as nucleosomes present formidable barriers to the passage of Pol II during transcriptional elongation [23] and global acetylation in transcribed regions is required for increased basal levels of transcription in yeast [24-26]. Acetylation of nucleosomal histones in the region of transcription start sites (TSSs) may stabilize the binding of chromatin remodeling factors to promoter regions [27] and/or destabilize nucleosome structure [28,29], leading to decreased nucleosome occupancy at TSSs that facilitates RNA Pol II binding [30]. H4K16ac has been shown to impact higher order chromatin structure and create an open, highly accessible environment [31,32] changing functional interactions between chromatin-associated proteins [32], and serving as a switch for altering chromatin from a repressive to a transcriptionally active state in yeast and humans [33].

Tip60 (Tat-interacting protein) acetylates histones H2A, H3 and H4 and plays a role in DNA repair [34]. In Drosophila, Tip60 acetylates nucleosomal phospho-H2Av and replaces it with an unmodified H2Av [16]. The acetylation of histone H4 by Esa1 (essential SAS2-related acetyltransferase1) is required for DNA repair in yeast [11] and thus a similar modification may function in mammalian cells. Depletion of human MOF (hMOF) in cells results in a corresponding H4K16ac loss [13,19,35-37] indicating that hMOF protein is the primary HAT responsible for histone H4 acetylation at K16. Recent studies have demonstrated that depletion of hMOF correlates with decreased DNA damage-induced activation of ATM and prevents ATM from phosphorylating downstream effectors, such as H2AX, p53 and CHK2 [13,37]. Furthermore, hMOF physically interacts with ATM [13,35] and hMOF binds to and acts synergistically with p53 to increase H4K16ac and target gene transcription in vitro [35]. In yeast, the presence of H4K16ac and H2A.Z synergistically prevent the ectopic propagation of heterochromatin in subtelomeric regions [33].

Consistent with these genetic and biochemical results, genome-wide location analysis of HAT binding in yeast demonstrated a good correlation with transcriptional activation [38]. Numerous combinatorial patterns of histone modifications, including those related to HATs, in human CD4^+^ T cells have been studied on a genome-wide scale [39,40] showing that modification patterns change during cell differentiation [41-43]. Furthermore, recent genome-wide binding analyses demonstrated that mouse Mof plays a critical role in the Nanog-dependent transcription network in mouse embryonic stem (ES) cells [44]. In the present studies we determine the genome-wide distribution of H4K16ac, the product of MOF acetylation, and compared it with MOF-dependent differential expression of genes in HEK293 human cancer cells. Surprisingly, the potential contribution to gene expression of H4K16ac in HEK293 cells is limited, as opposed to results from ES cell studies, suggesting that H4K16ac-dependent transcription regulation is cell type-dependent and that MOF may have other important roles in addition to transcriptional regulation through H4K14ac modification.

## Results and discussion

**Figure 1:**
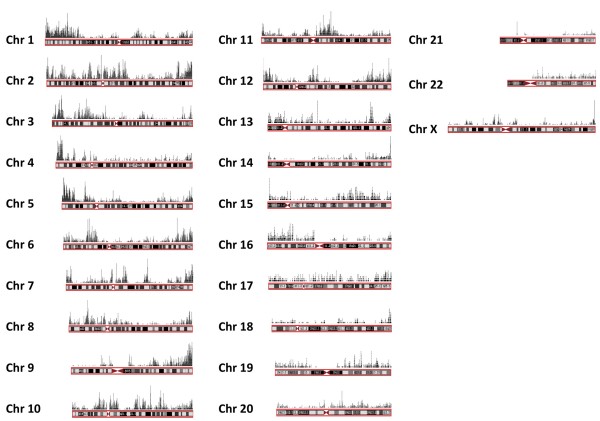
**Chromosomal distribution of H4K16ac sites.** HEK293 cell genomic DNA was isolated and subjected to ChIP-ChIP analysis using an antibody against H4K16ac and the NimbleGen Human Whole-Genome Tiling Array Set (see Materials and methods). Results are summarized for each chromosome with the bar-height representing the regional log_2_H4K14ac intensity >2-fold.

**Figure 2:**
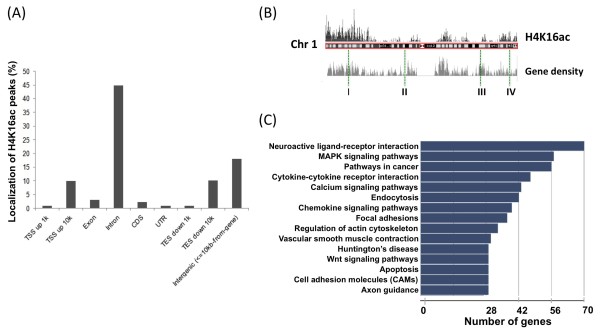
**Localization of H4K16ac peaks in the HEK293 genome.** (**A**) Genome-wide distribution of H4K16ac peaks. H4K14ac peak distribution was categorized as within exon, intron, coding and other genomic locations as described. Each bar represents the percentage of peaks in each category. (**B**) Correlation of H4K16ac peaks and gene density in human chromosome 1. The bar graph for H4K16ac density and the gene density map of human chromosome 1 are aligned. Although these two diagrams are well correlated (I and II), some regions show differences (III and IV). (**C**) Gene ontology for the genes enriched by H4K16ac ChIP. A total of 3,538 genes identified as H4K16ac positive genes are subjected to gene ontology analysis.

The histone code modification H4K16ac has been identified in various organisms as a probable marker of actively transcribed genes within euchromatin [45,46]. Acetylation of H4K16 is carried out by the MOF acetyltransferase and while the genomic distribution of MOF bound sites has been determined, a comprehensive mapping and analysis of H4K16ac sites has not been performed in human cells. To precisely identify all H4K16ac genomic locations, we performed chromatin immunoprecipitation (ChIP) from HEK293 cells with an H4K16ac specific antibody followed by hybridization of the co-precipitated DNA to NimbleGen whole-genome tiling arrays. Analysis of the tiling data identified 25,893 high confidence DNA associated regions [false discovery rate (FDR) <0.005] with an average length of 692 bases that were distributed unevenly throughout the genome (Figure 1). In general, there was a tendency for the regions near chromosome ends to have areas with high H4K16ac levels (41/46) while centromeric regions are largely free of H4K14ac (43/46). At a finer level, the majority (82%) of H4K16ac modification sites were located within genes: 45% within introns, 10% of sites 10 kb distal to annotated TSS, 10% near transcription end sites (TES) and a relatively small fraction of binding sites within exonic (3%) and coding (2%) regions (Figure 2A). Gene-poor regions, designated as intergenic regions, contain only 18% of H4K16ac sites. These results are generally consistent with the previously described analysis of Mof-associated DNA regions [44] in mouse embryonic stem cells (ESCs), where about 70% of sites were within genes, although a higher percentage of sites (~30%) were also localized in coding regions. A general correlation of H4K16ac distribution with gene density in human chromosome 1 was observed (regions I and II), although there were areas where the H4K16ac distribution did not correlate with gene density (regions III and IV) (Figure 2B).

To determine the role of H4K16ac histone modification in regulating gene expression, we analyzed the spectrum of H4K16ac sites genome-wide for locations within or near genes. We found 5002 of the 25,893 H4K16ac sites localized in 3538 unique genes within a region encompassing 10 kb upstream and downstream of annotated TSS. Gene ontology and KEGG pathways analysis using the GeneCodis2 web-based integrative knowledgebase [47] for the 3538 genes revealed the highest enrichment for neuroactive ligand-receptor interactions, MAPK signaling, focal adhesion, Wnt signaling, apoptosis and cancer pathways (Figure 2C).

**Figure 3:**
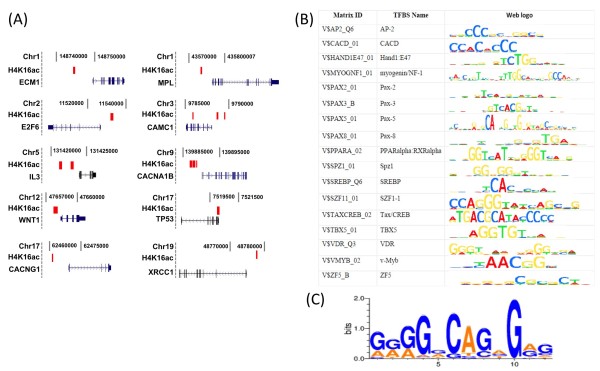
**Distribution of H4K16ac in genes and its associated sequences.** (**A**) Position of H4K16ac enriched fragments with respect to TSS (±10 kb of TSS; UCSC hg18) on 10 representative genes from different chromosomes is shown based on ChIP-chip data. (**B**) Transcription factor binding sites with significant enrichment (p-value <0.0005) within H4K16ac ChIP-chip peaks. Analysis was performed using TRANSFAC. (**C**) Sequence logo of the motif identified in H4K16ac-enriched DNA fragments.

Examples of H4K16ac peak localization in promoter/enhancer regions are shown in Figure 3A. The location in the chromosome, H4K16ac peaks (red) and gene name and exon/intron structure with transcription direction are shown. As a potential regulatory mechanism of gene expression, we next asked whether these H4K16ac peaks in or near genes overlap with transcription factor binding sites. Using position weight matrices representing DNA target sites for 184 transcription factors reported in the TRANSFAC database, we determined the number of these transcription factor-binding sites that were over-represented near H4K16 acetylation sites (relative to randomly selected control DNA fragments of the same size, see Materials and methods). We found 17 transcription factor-binding sites that were significantly associated with H4K16 acetylated regions compared to control (p < 0.0005, Figure 3B). Interestingly, all 17 transcription factor-binding sites were localized near promoters of genes implicated in cell cycle progression, proliferation and apoptosis. These observations support the previous studies describing the role of H4K16 acetylation in cell cycle regulation and cell growth [44,48]. The H4K16ac associated DNAs were also analyzed to determine whether a unique or novel DNA motif was over-represented in the bound DNAs. Analysis of all 25,893 binding peaks with the Gibbs motif sampler [49] identified a 12-mer G-rich sequence motif (Figure 3C). This motif is present at least once in more than 55% of all H4K16ac peaks identified in HEK293 cells. Since this motif is similar to the CTCF insulator binding (CCCTC-binding) protein motif (antisense), co-occupancy analysis of H4K16ac with CTCF DNA binding proteins was performed which revealed that 13,230 unique peaks, corresponding to 51% of H4K16ac peaks, contained at least one CTCF motif (data not shown).

**Figure 4:**
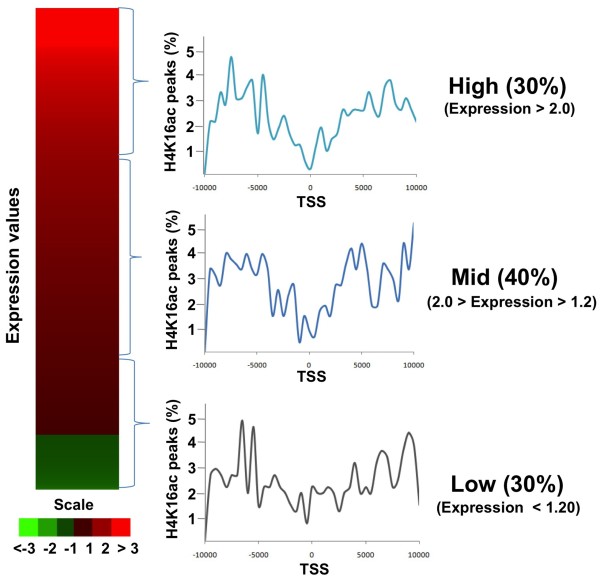
**Localization of H4K14ac peaks in genes.** Untreated HEK293 gene expression profiling data were used to determine the expression levels of entire genes and divided into three categories: top 30% high expression genes, bottom 30% low expression genes, middle 40% genes between. In each category, H4K16ac peak distribution with respect to TSS was determined and displayed.

Given that transcription factor binding motifs overlap with H4K16ac sites localized near promoters, it is possible that H4K16ac directly regulates gene expression through effects on transcription factors. Therefore, we also analyzed the H4K16ac peak distributions within ± 10 kb of gene TSS after dividing the genes into three categories based on HEK293 cell expression levels: top 30%, middle 40%, and bottom 30%. The HEK293 cell expression profile data were obtained from published microarray data analysis [50]. Within ±10 kb of TSS for the top 30% and middle 40% of expressed genes, the H4K16ac peak distributions were clearly different from those of MOF, which displayed a mono modal distribution at the TSS in mouse ESCs as well as human CD4^+^ T cells [44,51]. The H4K16ac density in the vicinity of TSS had a V-shape distribution (Figure 4), with a minimum in peak intensity near the TSS that was lower in high and middle level expression genes than in the low expression group but then increased rapidly on either side of high and middle expressed genes. Overall, the data indicate that H4K16ac containing histone deposition has a complex correlation with overall gene expression level in HEK293 cells as proposed earlier [33].

The H4K16ac distribution profile in HEK293 cells is similar to that observed in Drosophila and yeast [8,52,53]. On the Drosophila X chromosome, MOF displays a bimodal distribution, binding to the promoter and 3^′^ end regions of genes, whereas H4K16ac is preferentially located at the 3^′^ ends. Thus it seems likely that MOF distribution is not identical to that of H4K16ac and this tendency may be evolutionally conserved. MOF forms MOF-NSL complexes that are enriched at TSSs while a dosage compensation complex (DCC), also known as the MOF-MSL complex, is enriched at 3^′^ gene regions. Although MOF is enzymatically active in both complexes, H4K16ac is low at TSSs suggesting that the MOF-NSL complex may be either less efficient or subject to site or cell type specific inhibition. Alternatively, H4K16ac modified nucleosomes may be displaced up or down from TSS by alternative mechanisms involving transcription initiation or elongation. In support of a role for H4K16ac in transcription, a recent report demonstrated that the enhanced gene expression occurring during X chromosome dosage compensation in Drosophila is via H4K16ac-dependent enhancement of transcriptional elongation [53]. Perhaps in some HEK293 genes transcription levels are enhanced by the distribution of H4K16ac toward 3^′^ ends of genes, possibly as MOF-MSL or similar complexes support transcriptional elongation.

We next analyzed H4K16ac peaks within ±10 kb of TSS for genes whose expression was altered in MOF-depleted HEK293 cells using differential gene expression profiles obtained previously from control and MOF knockdown HEK293 cells [20,50]. Microarray profiling revealed that 1020 genes (up: 426 genes, down: 594 genes) were differentially expressed (fold change; >1.5) [50] which is smaller than the 4475 genes that changed more than 2-fold in expression [44,48] in MOF conditional knockout ESCs. Fewer genes may be differentially affected by MOF-depletion in HEK293 cells due to cell type differences; ESCs are rapidly growing pluripotent cells whereas HEK293 cells are kidney cell-derived cancer cells simply undergoing constant self-renewal. Thus, transcriptional regulation by MOF may play a more significant role in ESCs than HEK293 cells, even though MOF is essential in both situations for cell viability and proliferation [20,44]. Alternatively, the different results may be due to the fact that siRNA-mediated MOF depletion is only partial (~85%), since total depletion in 293 cells is lethal as is complete Mof gene knockout during mouse embryogenesis [20]. Induced Mof gene knockout in mouse ESCs by Cre expression eventually results in complete loss of Mof expression [44,48] and these cells therefore may have initiated changes related to cell death not observed during partial depletion.

**Figure 5:**
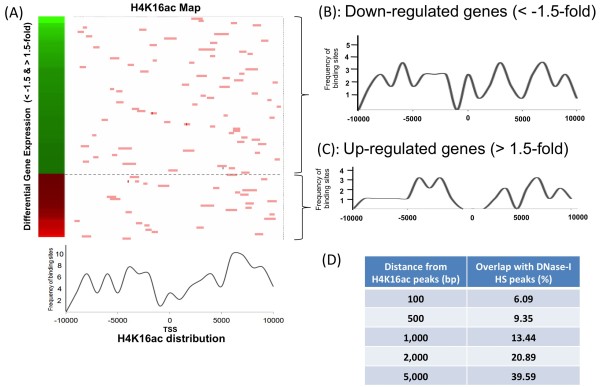
**H4K16ac distribution in genes differentially regulated by MOF status.** (**A**) H4K16ac peak distribution in differentially expressed genes. H4K14ac peaks are mapped within ±10 kb TSS with pink bars for each gene and the total H4K16ac distribution is shown in lower panel. Genes are aligned according to relative changes in expression of the transcript as determined by microarrays on targeted depletion of H4K16ac in HEK293 cells. (**B**) H4K16ac distribution in down-regulated genes (less than −1.5-fold). (**C**) H4K16ac distribution in up-regulated genes (more than 1.5-fold). (**D**) DNase I hypersensitive sites are poorly correlated with H4K16ac peaks in HEK293 cells. Total H4K16ac peaks were analyzed for DNase I hypersensitive sites. As shown in table, only 13.44% of H4K16ac peaks also contain DNase I hypersensitive sites within the region ±1 kb from the peak.

Finally, we determined the number of genes with altered expression following MOF depletion that also harbored H4K16ac peaks within ±10 kb of the TSS. Surprisingly, there were only 73 such genes, of which 26 were up- and 47 down-regulated (Figure 5A), a result significantly less than the 1295 up-regulated and 1557 down-regulated genes detected at Mof binding sites in mouse ESCs [44]. Ingenuity Pathway Analysis of the 73 genes indicated a majority were involved in cell cycle, DNA replication, recombination, repair, or cancer related pathways. The Nanog transcription network, which is a critical direct MOF-dependent transcription group in ESCs, was not enriched in HEK293 cells. Within the 73 gene subgroup, the H4K16ac distribution profiles for up- and down-regulated genes did not display any unique site differences that could account for differential expression (Figure 5B and 5C, respectively), though 5–10 kb upstream of the TSS, up-regulated genes had a slight plateau in distribution that was lower overall compared to the more irregular distribution seen in down-regulated genes. These results suggest that H4K16ac distribution does not correlate highly with transcriptional regulation in HEK293 cells, where it may also play a smaller role than it does in the critical self-renewal of mouse ESCs [44]. In yeast, SAS-I-dependent H4K16 acetylation is reported to be independent of transcription and histone exchange [52]. It is also demonstrated in yeast that H4K16ac is enriched in inactive genes [26]. Although, H4K16ac has been linked to X chromosome transcriptional elongation in Drosophila [53], MOF activates only a defined subset of promoters [54]. In mammalian cells, phosphorylation of H3 Ser10 during transcriptional activation leads to the recruitment of MOF, which in turn modulates transcription elongation via recruitment of BRD4 [2]. Indeed, MOF in Drosophila directly binds in a bimodal fashion to the promoters and 3^′^-end of dosage-compensated genes on the male X-chromosome and increases their expression [8]. Thus, MOF may have different roles in transcriptional regulation using different mechanisms that is organism and cell type specific. Indeed, MOF in Drosophila has been shown to have a critical role in DNA repair [55].

In summary, the present results imply that the role of H4K16ac in transcription regulation is cell type-dependent and possibly less important in differentiated cells where other epigenetic marks, such as DNA methylation, may override histone codes [56]. Interestingly, even though MOF dependent H4K16ac has only limited correlation with transcriptional regulation in HEK293 cells, it is still required for cell survival. Furthermore, only 13.44% of DNase I hypersensitive sites are localized within 1 kb of H4K16ac peaks in HEK293 cells (Figure 5D), suggesting that the majority of H4K16ac distributes to regions other than DNase I hypersensitive sites and loss of H4K16 acetylation, therefore, is unlikely to affect the global distribution of DNase I hypersensitive sites. Interestingly, it has been reported that the function of MOF is largely dependent on the activities of tumor suppressors [57]. Therefore, the critical role of MOF in transcriptional regulation may be in combination with other transcriptional factors, such as FOXP3 [57]. In other words, MOF dependent transcriptional regulation could be important only when cells are exposed to certain stimuli. Overall, our results suggest that although MOF may have a critical function in transcriptional regulation of some genes in specific cell types, additional functions other than transcriptional regulation underlie the requirement for MOF/H4K16ac for cell survival. These critical functions may be related to MOF-mediated acetylation of chromatin-associated proteins other than H4 or alterations of chromatin structure induced by such modifications that are not detectable by DNaseI sensitivity.

## Materials and methods

### Identification of H4K16ac peaks in HEK293 cells

HEK293 cells were cultured as described previously [13,20,50]. ChIP was performed in triplicate as described [50] using anti-H4K16ac antibody (EMD Millipore, Billerica, MA). Recovered DNA fragments were purified and ChIP-chip was performed in Roche NimbleGen, Inc. (Madison, WI). DNA without ChIP processing was used as a reference. The differential gene expression profile was obtained from GEO (accession number: GSE20193) [50].

Raw ChIP-chip fluorescence intensity data obtained from NimbleGen Human ChIP-chip 2.1 M Whole-Genome Tiling −10 Array Set (probe length: 50–75 mer, median probe spacing 100 bp) were analyzed using Roche NimbleScan 2.6 software. Settings included probe intensity ratio calculation between experimental (ChIP) sample and control (non-specific IgG) sample. Enrichment is quantified by probe intensity log ratios calculated as the base 2 logarithm of the ratio between the experimental and control samples. Using the default parameters (probe length = 50 bp; P start = 90; P step = 1; # step = 76; min probes > cutoff in peak = 4; when all probes in peak > cutoff = 2 and width of sliding window = 500 bp) peaks were identified at FDR <0.005. All coordinates in this manuscript are reported in hg18 (GEO accession number: GSE44254).

### Identification of an H4K16ac binding DNA motif

To identify DNA sequence motifs in the H4K16ac-bound regions detected by ChIP-chip, the sequences of all 25,893 H4K16ac-bound regions were retrieved from UCSC built hg18 and inputted into the Gibbs sampler motif finding program provided by CisGenome. The motif finder was run searching for motifs of 12 bp using 5,000 MCMC iterations and a score was produced for each motif.

### Transcription factor binding site and DNase I hypersensitive site enrichment analysis

For transcription factors enrichment analysis we first generated ten control sequences by randomly picking sequences of same size from the same chromosome. Both the peak sequences and control sequences were given as input into the Match tool to scan for enriched PWMs listed in TRANSFAC professional. The total occurrence of any given TFBS on each peak sequence was considered as the observed frequency. Similarly, the occurrence of a TFBS in control set sequences, gave the randomly expected frequency. The discrepancy between observed and expected frequency was evaluated by determining the statistical variable chi-square (χ^2^). The presence of CTCF motif within peak sequences (FDR < 0.005) was looked for the presence of 9-mer CTCF motif (AG[GA][GT]GG[CAT][GAT][CG] and their complementary sequence). We found 13,230 unique peaks (51% of overall peaks) with at least one 9-mer CTCF motif. DNase I hypersensitive sites data for HEK293T cells (a cell line derived from HEK293) were used for our correlation analysis obtained form: http://genome.ucsc.edu/cgi-bin/hgTrackUi?hgsid=302749441&c=chr6&g=wgEncodeOpenChromDnase.

## Competing interests

The authors declare that they have no competing interests.

## Authors’ contributions

NH, GGS, and TKP designed research; GGS performed ChIP-chip and MC network analyses; NH, PK, GGS, MC, CH, SC, and TKP performed experiments, provided reagents, and analyzed data; and NH, PK, GGS, MC, CH, KW, SC, and TKP reviewed data analyses and wrote the paper. All authors read and approved the final manuscript.

## References

[B1] Ruthenburg AJ Li H Milne TA Dewell S McGinty RK Yuen M Ueberheide B Dou Y Muir TW Patel DJ Allis CD Recognition of a mononucleosomal histone modification pattern by BPTF via multivalent interactions Cell 2011 145 692 706 10.1016/j.cell.2011.03.053 21596426 PMC3135172

[B2] Zippo A Serafini R Rocchigiani M Pennacchini S Krepelova A Oliviero S Histone crosstalk between H3S10ph and H4K16ac generates a histone code that mediates transcription elongation Cell 2009 138 1122 1136 10.1016/j.cell.2009.07.031 19766566

[B3] Conrad T Akhtar A Dosage compensation in Drosophila melanogaster: epigenetic fine-tuning of chromosome-wide transcription Nat Rev Genet 2011 13 123 134 22251873 10.1038/nrg3124

[B4] Gelbart ME Kuroda MI Drosophila dosage compensation: a complex voyage to the X chromosome Development 2009 136 1399 1410 10.1242/dev.029645 19363150 PMC2674252

[B5] Lucchesi JC The structure-function link of compensated chromatin in Drosophila Curr Opin Genet Dev 2009 19 550 556 10.1016/j.gde.2009.10.004 19880310 PMC2787700

[B6] Prestel M Feller C Straub T Mitlohner H Becker PB The activation potential of MOF is constrained for dosage compensation Mol Cell 2010 38 815 826 10.1016/j.molcel.2010.05.022 20620953

[B7] Conrad T Cavalli FM Holz H Hallacli E Kind J Ilik I Vaquerizas JM Luscombe NM Akhtar A The MOF chromobarrel domain controls genome-wide H4K16 acetylation and spreading of the MSL complex Dev Cell 2012 22 610 624 10.1016/j.devcel.2011.12.016 22421046

[B8] Kind J Vaquerizas JM Gebhardt P Gentzel M Luscombe NM Bertone P Akhtar A Genome-wide analysis reveals MOF as a key regulator of dosage compensation and gene expression in Drosophila Cell 2008 133 813 828 10.1016/j.cell.2008.04.036 18510926

[B9] Mendjan S Taipale M Kind J Holz H Gebhardt P Schelder M Vermeulen M Buscaino A Duncan K Mueller J Nuclear pore components are involved in the transcriptional regulation of dosage compensation in Drosophila Mol Cell 2006 21 811 823 10.1016/j.molcel.2006.02.007 16543150

[B10] Raja SJ Charapitsa I Conrad T Vaquerizas JM Gebhardt P Holz H Kadlec J Fraterman S Luscombe NM Akhtar A The nonspecific lethal complex is a transcriptional regulator in Drosophila Mol Cell 2010 38 827 841 10.1016/j.molcel.2010.05.021 20620954

[B11] Bird AW Yu DY Pray-Grant MG Qiu Q Harmon KE Megee PC Grant PA Smith MM Christman MF Acetylation of histone H4 by Esa1 is required for DNA double-strand break repair Nature 2002 419 411 415 10.1038/nature01035 12353039

[B12] Bone JR Lavender J Richman R Palmer MJ Turner BM Kuroda MI Acetylated histone H4 on the male X chromosome is associated with dosage compensation in Drosophila Genes Dev 1994 8 96 104 10.1101/gad.8.1.96 8288132

[B13] Gupta A Sharma GG Young CS Agarwal M Smith ER Paull TT Lucchesi JC Khanna KK Ludwig T Pandita TK Involvement of human MOF in ATM function Mol Cell Biol 2005 25 5292 5305 10.1128/MCB.25.12.5292-5305.2005 15923642 PMC1140595

[B14] Hilfiker A Hilfiker-Kleiner D Pannuti A Lucchesi JC mof, a putative acetyl transferase gene related to the Tip60 and MOZ human genes and to the SAS genes of yeast, is required for dosage compensation in Drosophila EMBO J 1997 16 2054 2060 10.1093/emboj/16.8.2054 9155031 PMC1169808

[B15] Kimura A Matsubara K Horikoshi M A decade of histone acetylation: marking eukaryotic chromosomes with specific codes J Biochem 2005 138 647 662 10.1093/jb/mvi184 16428293

[B16] Kusch T Florens L Macdonald WH Swanson SK Glaser RL Yates JR 3rd Abmayr SM Washburn MP Workman JL Acetylation by Tip60 is required for selective histone variant exchange at DNA lesions Science 2004 306 2084 2087 10.1126/science.1103455 15528408

[B17] Sterner DE Berger SL Acetylation of histones and transcription-related factors Microbiol Mol Biol Rev 2000 64 435 459 10.1128/MMBR.64.2.435-459.2000 10839822 PMC98999

[B18] Suka N Luo K Grunstein M Sir2p and Sas2p opposingly regulate acetylation of yeast histone H4 lysine16 and spreading of heterochromatin Nat Genet 2002 32 378 383 10.1038/ng1017 12379856

[B19] Taipale M Rea S Richter K Vilar A Lichter P Imhof A Akhtar A hMOF histone acetyltransferase is required for histone H4 lysine 16 acetylation in mammalian cells Mol Cell Biol 2005 25 6798 6810 10.1128/MCB.25.15.6798-6810.2005 16024812 PMC1190338

[B20] Gupta A Guerin-Peyrou TG Sharma GG Park C Agarwal M Ganju RK Pandita S Choi K Sukumar S Pandita RK The mammalian ortholog of Drosophila MOF that acetylates histone H4 lysine 16 is essential for embryogenesis and oncogenesis Mol Cell Biol 2008 28 397 409 10.1128/MCB.01045-07 17967868 PMC2223300

[B21] Thomas T Dixon MP Kueh AJ Voss AK Mof (MYST1 or KAT8) is essential for progression of embryonic development past the blastocyst stage and required for normal chromatin architecture Mol Cell Biol 2008 28 5093 5105 10.1128/MCB.02202-07 18541669 PMC2519697

[B22] Kumar R Hunt CR Gupta A Nannepaga S Pandita RK Shay JW Bachoo R Ludwig T Burns DK Pandita TK Purkinje cell-specific males absent on the first (mMof) gene deletion results in an ataxia-telangiectasia-like neurological phenotype and backward walking in mice Proc Natl Acad Sci USA 2011 108 3636 3641 10.1073/pnas.1016524108 21321203 PMC3048124

[B23] Orphanides G LeRoy G Chang CH Luse DS Reinberg D FACT, a factor that facilitates transcript elongation through nucleosomes Cell 1998 92 105 116 10.1016/S0092-8674(00)80903-4 9489704

[B24] Govind CK Zhang F Qiu H Hofmeyer K Hinnebusch AG Gcn5 promotes acetylation, eviction, and methylation of nucleosomes in transcribed coding regions Mol Cell 2007 25 31 42 10.1016/j.molcel.2006.11.020 17218269

[B25] Vogelauer M Wu J Suka N Grunstein M Global histone acetylation and deacetylation in yeast Nature 2000 408 495 498 10.1038/35044127 11100734

[B26] Natsume-Kitatani Y Shiga M Mamitsuka H Genome-wide integration on transcription factors, histone acetylation and gene expression reveals genes co-regulated by histone modification patterns PLoS One 2011 6 e22281 10.1371/journal.pone.0022281 21829453 PMC3146477

[B27] Hassan AH Neely KE Workman JL Histone acetyltransferase complexes stabilize swi/snf binding to promoter nucleosomes Cell 2001 104 817 827 10.1016/S0092-8674(01)00279-3 11290320

[B28] Boeger H Griesenbeck J Strattan JS Kornberg RD Nucleosomes unfold completely at a transcriptionally active promoter Mol Cell 2003 11 1587 1598 10.1016/S1097-2765(03)00231-4 12820971

[B29] Reinke H Horz W Histones are first hyperacetylated and then lose contact with the activated PHO5 promoter Mol Cell 2003 11 1599 1607 10.1016/S1097-2765(03)00186-2 12820972

[B30] Schones DE Cui K Cuddapah S Roh TY Barski A Wang Z Wei G Zhao K Dynamic regulation of nucleosome positioning in the human genome Cell 2008 132 887 898 10.1016/j.cell.2008.02.022 18329373 PMC10894452

[B31] Bell O Schwaiger M Oakeley EJ Lienert F Beisel C Stadler MB Schubeler D Accessibility of the Drosophila genome discriminates PcG repression, H4K16 acetylation and replication timing Nat Struct Mol Biol 2010 17 894 900 10.1038/nsmb.1825 20562853

[B32] Shogren-Knaak M Ishii H Sun JM Pazin MJ Davie JR Peterson CL Histone H4-K16 acetylation controls chromatin structure and protein interactions Science 2006 311 844 847 10.1126/science.1124000 16469925

[B33] Shia WJ Li B Workman JL SAS-mediated acetylation of histone H4 Lys 16 is required for H2A.Z incorporation at subtelomeric regions in Saccharomyces cerevisiae Genes Dev 2006 20 2507 2512 10.1101/gad.1439206 16980580 PMC1578674

[B34] Ikura T Ogryzko VV Grigoriev M Groisman R Wang J Horikoshi M Scully R Qin J Nakatani Y Involvement of the TIP60 histone acetylase complex in DNA repair and apoptosis Cell 2000 102 463 473 10.1016/S0092-8674(00)00051-9 10966108

[B35] Dou Y Milne TA Tackett AJ Smith ER Fukuda A Wysocka J Allis CD Chait BT Hess JL Roeder RG Physical association and coordinate function of the H3 K4 methyltransferase MLL1 and the H4 K16 acetyltransferase MOF Cell 2005 121 873 885 10.1016/j.cell.2005.04.031 15960975

[B36] Smith ER Cayrou C Huang R Lane WS Cote J Lucchesi JC A human protein complex homologous to the Drosophila MSL complex is responsible for the majority of histone H4 acetylation at lysine 16 Mol Cell Biol 2005 25 9175 9188 10.1128/MCB.25.21.9175-9188.2005 16227571 PMC1265810

[B37] Sykes SM Mellert HS Holbert MA Li K Marmorstein R Lane WS McMahon SB Acetylation of the p53 DNA-binding domain regulates apoptosis induction Mol Cell 2006 24 841 851 10.1016/j.molcel.2006.11.026 17189187 PMC1766330

[B38] Shahbazian MD Grunstein M Functions of site-specific histone acetylation and deacetylation Annu Rev Biochem 2007 76 75 100 10.1146/annurev.biochem.76.052705.162114 17362198

[B39] Barski A Cuddapah S Cui K Roh TY Schones DE Wang Z Wei G Chepelev I Zhao K High-resolution profiling of histone methylations in the human genome Cell 2007 129 823 837 10.1016/j.cell.2007.05.009 17512414

[B40] Wang Z Zang C Rosenfeld JA Schones DE Barski A Cuddapah S Cui K Roh TY Peng W Zhang MQ Zhao K Combinatorial patterns of histone acetylations and methylations in the human genome Nat Genet 2008 40 897 903 10.1038/ng.154 18552846 PMC2769248

[B41] Cui K Zang C Roh TY Schones DE Childs RW Peng W Zhao K Chromatin signatures in multipotent human hematopoietic stem cells indicate the fate of bivalent genes during differentiation Cell Stem Cell 2009 4 80 93 10.1016/j.stem.2008.11.011 19128795 PMC2785912

[B42] Wang Z Schones DE Zhao K Characterization of human epigenomes Curr Opin Genet Dev 2009 19 127 134 10.1016/j.gde.2009.02.001 19299119 PMC2699568

[B43] Wei G Wei L Zhu J Zang C Hu-Li J Yao Z Cui K Kanno Y Roh TY Watford WT Global mapping of H3K4me3 and H3K27me3 reveals specificity and plasticity in lineage fate determination of differentiating CD4+ T cells Immunity 2009 30 155 167 10.1016/j.immuni.2008.12.009 19144320 PMC2722509

[B44] Li X Li L Pandey R Byun JS Gardner K Qin Z Dou Y The histone acetyltransferase MOF is a key regulator of the embryonic stem cell core transcriptional network Cell Stem Cell 2012 11 163 178 10.1016/j.stem.2012.04.023 22862943 PMC3413170

[B45] Millar CB Kurdistani SK Grunstein M Acetylation of yeast histone H4 lysine 16: a switch for protein interactions in heterochromatin and euchromatin Cold Spring Harb Symp Quant Biol 2004 69 193 200 10.1101/sqb.2004.69.193 16117649

[B46] Vaquero A Scher MB Lee DH Sutton A Cheng HL Alt FW Serrano L Sternglanz R Reinberg D SirT2 is a histone deacetylase with preference for histone H4 Lys 16 during mitosis Genes Dev 2006 20 1256 1261 10.1101/gad.1412706 16648462 PMC1472900

[B47] Carmona-Saez P Chagoyen M Tirado F Carazo JM Pascual-Montano A GENECODIS: a web-based tool for finding significant concurrent annotations in gene lists Genome Biol 2007 8 R3 10.1186/gb-2007-8-1-r3 17204154 PMC1839127

[B48] Megee PC Morgan BA Mittman BA Smith MM Genetic analysis of histone H4: essential role of lysines subject to reversible acetylation Science 1990 247 841 845 10.1126/science.2106160 2106160

[B49] Lawrence CE Altschul SF Boguski MS Liu JS Neuwald AF Wootton JC Detecting subtle sequence signals: a Gibbs sampling strategy for multiple alignment Science 1993 262 208 214 10.1126/science.8211139 8211139

[B50] Sharma GG So S Gupta A Kumar R Cayrou C Avvakumov N Bhadra U Pandita RK Porteus MH Chen DJ MOF and histone H4 acetylation at lysine 16 are critical for DNA damage response and double-strand break repair Mol Cell Biol 2010 30 3582 3595 10.1128/MCB.01476-09 20479123 PMC2897562

[B51] Wang Z Zang C Cui K Schones DE Barski A Peng W Zhao K Genome-wide mapping of HATs and HDACs reveals distinct functions in active and inactive genes Cell 2009 138 1019 1031 10.1016/j.cell.2009.06.049 19698979 PMC2750862

[B52] Heise F Chung HR Weber JM Xu Z Klein-Hitpass L Steinmetz LM Vingron M Ehrenhofer-Murray AE Genome-wide H4 K16 acetylation by SAS-I is deposited independently of transcription and histone exchange Nucleic Acids Res 2012 40 65 74 10.1093/nar/gkr649 21908408 PMC3245914

[B53] Larschan E Bishop EP Kharchenko PV Core LJ Lis JT Park PJ Kuroda MI X chromosome dosage compensation via enhanced transcriptional elongation in Drosophila Nature 2011 471 115 118 10.1038/nature09757 21368835 PMC3076316

[B54] Feller C Prestel M Hartmann H Straub T Soding J Becker PB The MOF-containing NSL complex associates globally with housekeeping genes, but activates only a defined subset Nucleic Acids Res 2012 40 1509 1522 10.1093/nar/gkr869 22039099 PMC3287193

[B55] Bhadra MP Horikoshi N Pushpavallipvalli SN Sarkar A Bag I Krishnan A Lucchesi JC Kumar R Yang Q Pandita RK The role of MOF in the ionizing radiation response is conserved in Drosophila melanogaster Chromosoma 2012 121 79 90 10.1007/s00412-011-0344-7 22072291 PMC4151556

[B56] Jones PA Functions of DNA methylation: islands, start sites, gene bodies and beyond Nat Rev Genet 2012 13 484 492 10.1038/nrg3230 22641018

[B57] Katoh H Qin ZS Liu R Wang L Li W Li X Wu L Du Z Lyons R Liu CG FOXP3 orchestrates H4K16 acetylation and H3K4 trimethylation for activation of multiple genes by recruiting MOF and causing displacement of PLU-1 Mol Cell 2011 44 770 784 10.1016/j.molcel.2011.10.012 22152480 PMC3243051

